# Supplementary material for: Unifying information theory and machine learning in a model of electrode discrimination in cochlear implants
Source: PLoS One. 2021 Sep 20;16(9):e0257568. doi: 10.1371/journal.pone.0257568 (PMC8451994; doi:10.1371/journal.pone.0257568)
Supplement: Attachment — Submitted filename: RebuttalLetter_PONE-D-21-01888_XiaoGAO.pdf [file pone.0257568.s002.pdf]

PONE-D-21-01888 Review

Title: Unifying information theory and machine learning in a model of electrode discrimination in cochlear implants

Reviewer 1

General comments:

The authors present a method to model the effect of several of the most important parameters on CI electrode discrimination. In this method they combine an information theory approach that computes the mutual information between input and output with a classifier that predicts the stimulated electrode from a given nerve fiber activation pattern. The method is described clearly and the manuscript is written in an intelligible fashion. Even though both, the classifier and the information theory approach are described in their own sections, the manuscript would benefit from a more detailed description of the combination of the two methods.

Author response:

We thank for the reviewer for their comments on the manuscript. We have described the combination of the two methods in the Section "The framework for electrode discrimination" with Fig. 3 showing how correct classification and mutual information are obtained.

Author action:

In the revised version, we changed the Section title "The framework for electrode discrimination" to "Unifying information theory and machine learning in the framework for electrode discrimination", and we have added detailed explanation on how these two methods are combined in the same framework. We hope this will assist readers to understand how we combine the two methods.

1. In the results section, the authors could elaborate more on the discrepancies between the results. Why are there substantial differences between the mutual information and the correct electrode classification rate for the same set of parameters?

   Author response:

   Mutual information provides a metric of correlation between input signals and consequent response; therefore, it is an useful metric for studying how adding more electrodes affects performance in a model of cochlear implants and predicting the optimal number of electrodes for a given parameter set. Correct classification rate, on the other hand, measures the accuracy of electrode discrimination at each electrode location. In our system, correct classification rates quantify the probabilities that each electrode can be correctly discriminated whereas mutual information is the measurement of the "information content" between a stimulated electrode and response of auditory nerve fibers. Therefore, increased mutual information is usually associated with improved perception and the maximum mutual information can indicate the number of electrodes that leads to optimal perception. In the manuscript, we showed a rapid increase of mutual information from 5 electrodes to around 12 electrodes; however, more than 11 electrodes only led to a marginal change in mutual information, whereas fewer than 12 electrodes led to a sharp drop in the total correct classification rate (Fig. 4). This indicates that hearing perception would not significantly improve for more than 12 electrodes. This finding is consistent with clinical studies.

   In terms of the differences between the mutual information and the correct electrode classification rate for the same set of parameters, we have explained why the correct classification rate information for 11 electrodes and 60 electrodes are different while mutual information for the two arrays is approximately the same (Section "Correct classification rate vs.

mutual information”, Fig. 5). This indicates that the level of perception of 11-electrode and 60-electrode arrays are similar.

Author action:

In the revised version, we have added explanation in the Results section of how mutual information and correct classification are related to help readers better relate mutual information to correct classification.

2. Minor remarks:
   line 33: the word order seems wrong
   line 169: missing word or incorrect grammar
   line 192: reference to the wrong figure (should be 4(a))
   line 300: "model" instead of "mode" and "have" instead of "has"
   Fig. 7: The x axis label does not match the figure description.
   Author action:
   In the revised version, we have amended these errors and typos.

Reviewer 2

1. Lack of validation of the model. I would suggest that the authors make an effort comparing in more detail the predictions of the model with more detailed data in the literature. For instance one could compare published data on electrode discrimination with different electrode distances and compare it to the predictions of the model. But this is just an example, the authors should make an effort in looking for the exact data that can validate each of the simulations (Figures) of the model. This is a journal paper that extends previous works from the authors, so now it is an opportunity to demonstrate that this model/framework can at least reproduce qualitatively electrode discrimination tasks. For example, if you refer to virtual channel discrimination you should provide data about virtual channel discrimination and demonstrate that your model obtains the same scores as the data.
   Author response:
   This manuscript focuses on unifying information theory and machine learning in an electrode discrimination model and showing that the model enables quantitative study of electrode discrimination. It can be extended to further study of hearing performance with cochlear implants at the individual level, which we intend to undertake as an extensive next step. However, we agree that the manuscript would benefit from comparison to clinical studies.

   Author action:
   In the revised version, we have added qualitative comparison between our results and clinical electrode discrimination studies (In Section Results, Correct classification rate vs. mutual information and Parameter sensitivity). We have also discussed the flexibility of the model structure and the potential to extend the model to study hearing performance at the individual level. We hope this can assist readers to see the importance of developing such a model.

2. More importantly, the aforementioned articles are examples of how modeling results can be clearly presented to a broader audience and connected to psychophysical measures. In the article under review, the analyses presented do not sufficiently consider neural response, but instead jump to digested metrics of mutual information and predicted discrimination based on machine learning. Doing so obscures the functionality of the model. I recommend that the authors analyze and describe the modeled neural response as a first-level of characterization. This is essential to demonstrating that the neural response accounts for known physiological

data associated with current spread. The closest to such that I could find from the authors' prior work is their physical review manuscript from 2014 (fig 4), but converting to average rate of discharge would be a better starting point for characterizing the model.

Author response:

The model of single auditory nerve fiber action potential generation and activation pattern generation are described in detail in McDonnell et al. 2010 and Gao et al. 2014. Therefore, we did not include these details in this manuscript. In this manuscript, we included a plot in the Methods section of how we generated the activation pattern for a given stimulated electrode. We explained how we modelled single auditory nerve fiber action potential generation and generated activation patterns. However, we agree with the reviewer that we should include some descriptions of neural response to make it easier to connect our modelling results to electrode discrimination measurements.

Author action:

In the revised version, we have added subplots in Fig 2 to show how the simulated activation patterns change when changing the location of stimulated electrode. By adding these plots as example cases, we hope this can assist readers to correlate the neural response of our electrode discrimination model better.

3. Some mathematical formulation is not clear. When describing the neural network you say that you train the Wout coefficients, but what happens to the Win coefficients? Are these not trained? If not, why not?

Author response:

The structure of the network used in this paper was introduced by McDonnell et al. 2015 PLoS One as a variation of the Extreme Learning Machine algorithm. The weights from the input to the hidden layer neurons are randomly initialized and are fixed thereafter (i.e., they are not trained). We chose this network structure because of its lower training complexity. The performance of the Extreme Learning Machine algorithm was tested on the MNIST test set and the results were equivalent or superior to the original results achieved by CNN and DBN and are achieved with significantly lower network and training complexity.

Author action:

In the revised version, we have added an explanation about why we chose this network structure and explained why Win is not trained in Methods section, Quantifying electrode discrimination ability.

4. Across the manuscript you use the term “performance of cochlear implants”. I would suggest to substitute these terms by “performance of cochlear implant users”.

Author response:

We agree that we need to be more specific about “performance of cochlear implants”. However, instead of using “performance of cochlear implant users”, we think that “performance of the cochlear implant model” is more suitable since this is a modelling study.

Author action:

In the revised version, we have changed “performance of cochlear implants” to “performance of the cochlear implant model”.

5. You did a nice sensitivity analysis of your model for some parameters. However, what is the effect of choosing different values for parameters of your network? Size of the hidden layer? Type of activation functions, etc?

Author response:

In the Discussion and Conclusion, we included a section "The choice of classifier" where we explained why we chose the neural network structure and the size of the hidden layer. We stated that we chose a single hidden layer because electrode discrimination has less complexity compared to many other tasks and we have achieved good prediction with a single hidden layer. We chose 1500 hidden layer units because optimal classification was found to occur with this number of units. In terms of the activation function, the absolute value function was used in this work; other nonlinear activation functions may also be used effectively.

Author action:

In the revised version, we have added in the Section "The choice of classifier" more explanation on why we chose the network structure and which type of activation function was chosen. We have also added discussion that the choices of network structure can be changed depending on the speech recognition tasks we choose to simulate.

Detailed comments

Abstract

"limit performance of cochlear implants" --> "Limit performance of cochlear implant users"

"It provide insights it provide insights" --> "it provides insights"

Author action:

In the revised version, we have amended the errors and typos.

Introduction

Third line: What do you mean here with extracellular electrodes? I agree the electrodes are extracellular but this definition seems a bit out of cochlear implant context.

Author action:

In the revised version, we use electrodes other than extracellular electrodes.

Last line of the first paragraph: Here you give three references at the very end [3,6,7]. Please provide detailed reference to each of your statements i); ii) and iii). Please check if results in the literature are statistically significant for each of the factors.

Author action:

In the revised version, we have added detailed reference for each statement.

First line second paragraph: "performance of cochlear implant" ◊ "performance of cochlear implant users". Please correct this terminology across the whole manuscript where it is used multiple times. Second line of the second paragraph: Here you state "… electrode (discrimination) has been used as a primary psychophysical measurement to assess the performance cochlear implants" I disagree with this statement. As far as I know there are no studies showing a significant correlation between electrode discrimination and speech understanding performance. Otherwise please provide these references. I agree though that many researchers investigated electrode discrimination performance as you mention in the following lines, and that this measure may impact speech understanding performance. But it is not a primary measurement, for sure not used in clinical environments.

Author response:

We agree that electrode discrimination is not the primary measurement for evaluating speech understanding in clinical environments. However, one of the factors that may directly affect speech perception ability with a multichannel implant is the ability to discriminate stimulation on different electrodes. For example, Henry et al. 2000 JASA showed that "speech information in the low to

medium frequencies is more difficult for implantees to perceive, that this difficulty is correlated with the difficulty in discriminating electrode place in the presence of random loudness variations, and that fine spectral discrimination may be relatively more important in the vowel-formant regions than in higher frequency region". Thus, to quantitatively study the variability of speech understanding at the individual level, we think it is important to study electrode discrimination.

To better understand the variability of speech recognition, we think it is important to model both electrode discrimination and speech recognition. However, it is beyond the scope of this paper. We think this is an important next step in the future development of the model.

Author action:

In the revised version, we have added in the Introduction how electrode discrimination and speech recognition are correlated and the importance of studying electrode discrimination (with references). In the Discussion and Conclusion, we also highlighted the need to extend the model to also model speech recognition along with electrode discrimination for better understanding of the variability of speech understanding at the individual level.

Page 2 Line 4: Rewrite as "A focus of recent studies is to investigate how …" Actually the two parts of this sentence seem to be a bit disconnected : First part is about "insertion depth" second part is about "virtual channels". Divide the sentence into two and expand what you are trying to convey here.

Author action:

In the revised version, we have reworded the sentence to make it easier to read.

Page 2 second paragraph Line 1: "Mathematical and computational model" ◊ "Mathematical and computational models"

Author action:

In the revised version, we have corrected this error.

Page 2 second paragraph Line 2: Here you cite [15] and [16] but there are many models out there. I would suggest to extend to recent models by Kalkman et al. (2015), Nogueira et al. (2016) and Bai et al. (2019).

Author action:

In the revised version, we have reviewed the models by Nogueira et al. 2016 as suggested by the reviewer. Bai et al. 2019 and Kalkman et al. 2015 are already in our reference list; we added them in the Introduction.

Page 2 second paragraph Line 6: What is a Psychophysical cochlear implant model? Please define.

Author action:

Psychophysical cochlear implant models are models of psychophysical responses to cochlear implant stimulation. In the revised version, we added an explanation of a psychophysical cochlear implant model.

Page 2 second paragraph Line 12: "[25] demonstrated …" Here you can refer to more recent models by Jürgens et al 2018 PlosOne.

Author action:

In the revised version, we have referred to the Jürgens et al. 2018 model as suggested.

Page 3 Caption of Figure 1: Specify the units after 5pi

Author action:

In the revised version, we have changed 2pi and 5pi to 2pi radians and 5pi radians.

Page 3 Equation 3: How accurate is this simple model of auditory nerve spiking? For example how accurate can you reproduce auditory nerve activity in comparison to data or to other models such as Joshi or Litvak.

Author response:

The stochastic model for action potential generation that we used (Bruce et al. 99) has been well received in the field and proven to be a robust and useful model of auditory nerve spiking. We agree that other models may have better accuracy and may be beneficial to choose when extending our model to a user-specific model.

Author action:

In the revised version, we have added explanation in the Methods section of why we chose this simple model for action potential generation. In the Discussion and Conclusion, we also highlighted the potential importance of adopting a more accurate model of auditory nerve spiking in the next stage of our model development.

P of the last paragraph “predication” should be “prediction”?

Author action:

In the revised version, we have corrected this typo.

Page 5 Line 1: “…we defined a vector, T, where each …” instead of a vector, isn’t T a matrix?

Author action:

We agree that T is a matrix and we have corrected this in the revised version.

Page 5 second line after Equation 7. I would suggest to remove “neuron” and just use the term “activation function” to make sure that the rather does not conus the activation function with Equation 3.

Author action:

In the revised version, we removed “neuron” to avoid the confusion.

Page 5 first line after equation 8: Here you state that Wout is obtained by training. What happens with Win?

Author response:

The input weights are random, as in McDonnell 2015 PLoS One and Gao 2015 IEEE TBME. Output weights Wout are obtained by minimizing the mean square error between V and Z. This is obtained using the closed form expression for such linear regression problems,

$$\boldsymbol{W}_{out} = \boldsymbol{V}\boldsymbol{A}_{train}^{\mathrm{T}}(\boldsymbol{A}_{train}\boldsymbol{A}_{train}^{\mathrm{T}})^{-1}$$

Author action:

In the revised version, we have added this explanation to make our description of the neural network clearer to the readers.

Page 5 first line Which activation function did you use in the output layer of your network. I think it is not specified.

Author response:

In this paper, the absolute value function is used for neuron activations. Other nonlinear activation functions may also be used effectively.

Author action:

In the revised version, we have added what activation function we chose.

Results

Page 6 first line: Here again use “cochlear implant users”

Author action:

In the revised version, we changed “performance of cochlear implants” to “performance of the cochlear implant model”.

Page 6 last line first paragraph: “400 tests” How many for each electrode?

Author response:

In the manuscript, Page 6 first paragraph, we have mentioned that “in order to estimate the mutual information, the correct classification rate and mutual information is calculated from 400 tests for each possible electrode choice.” Thus, it is 400 tests per electrode.

Author action:

In the revised version, we have reworded this sentence to make it clear for readers.

Page 6: “we find higher correct classification rate for a longer electrode array (alpha = 3pi). Is this improvement caused by the electrode array being longer or just by the fact that the electrode spacing is longer? In other words, you should show that a short array with increased electrode spacing results in worse performance.

Author response:

In our paper Gao et al. 2015 IEEE TBME, we discussed correct classification versus electrode spacing. We showed that when the distance between adjacent electrodes is less than ~0.7mm, a longer electrode array always has higher correct classification rate than a short electrode array.

Author action:

In the revised version, we have added this discussion and referred to our published paper for more details.

Page 7: Caption of Figure 5: Define electrode index.

Author action:

We number the electrodes from the basal end to the apical end in sequence. In the revised version, we have added this explanation.

Page 8_1: First line second paragraph: “We notice that only small changes are shown when varying electrode-to fiber.. .. this is because electrode discrimination is not as sensitive as speech recognition to changes in r” This statement seems a bit strange. You should provide references and give more detail why this happens. If r is increased electric spread will increase? Why are you not observing a worsening in electrode discrimination? Maybe you can check data comparing electrode discrimination with modiolar vs lateral wall electrodes? Or electrode discrimination with positioner vs no-positioner. Your model needs more data to be validated in general, at least qualitatively.

Author response:

In terms of why only marginal changes are shown when varying r, we think one of the reasons is that we modelled electrode discrimination rather than speech recognition. Electrode discrimination is not the ideal test for showing how the electrode-to-fiber distance affects hearing performance. Previous studies have suggested that up to 120 spectral channels can be distinguished via simultaneous virtual channel stimulation (Donaldson et al. 2005, Landsberger et al. 2012). However, speech recognition ability is not significantly improved even if more than 22 individual spectral channels can be distinguished. Therefore, we think speech recognition should be more suitable to test how electrode-to-fiber distance affects hearing performance.

Author action:

In the revised version, we have explained in the Results section why our model only shows slight changes when varying some of the parameters in the model, and we qualitatively compared our results to clinical studies. We also added discussion to emphasize the importance of updating each individual component according to clinical studies and model speech recognition in the future.

Page 8_2 second paragraph last line: "previous studies have suggested 120 spectral channels.." I don't understand the connection between this sentence and the previous ones. In you model you did not model virtual channels. Please model virtual channels or be more specific with the data that you use to validate your model.

Author action:

The reason that we cited Donaldson et al. 2005, Landsberger et al. 2012 was explained in the previous answer. We have reworded this paragraph to make it clearer to the readers that we did not model virtual channels.

Page 8_3: Last line of the second last paragraph: Over-training or Over-fitting?

Author response:

In the revised version, we have corrected this error.

Page 9: Last paragraph: I should be "to model electrode … " In this section again use "performance of cochlear implant users"

Author action:

We have changed "performance of cochlear implants" to "performance of the cochlear implant model" in the revised version.

In general in the results section I miss more comparison to studies in humans comparing your model predictions with real data, at least qualitatively.

Author response:

We have added qualitative comparison to real data in the Results section.
